# Topical Management of Wound: A Narrative Review of Cadexomer Iodine Ointment Versus Povidone Iodine Ointment

**DOI:** 10.7759/cureus.24598

**Published:** 2022-04-29

**Authors:** Shubham Gupta, Sangita Shinde, Raju K Shinde

**Affiliations:** 1 General Surgery, Datta Meghe Institute of Medical Sciences (Deemed to be University), Wardha, IND; 2 Pharmacology, Datta Meghe Institute of Medical Sciences (Deemed to be University), Wardha, IND

**Keywords:** topical ointments, cadexomer iodine, povidone iodine, wound, ulcer

## Abstract

Several iodine formulations have been used for wound care for ages, but still there exist a number of controversial issues regarding their uses in the present era. Many published studies are available for both povidone iodine (PI) and cadexomer iodine (CI) with conflicting outcomes due to different preparations used and different study types. PI has a broad spectrum of activity including antiseptic properties, anti-inflammatory properties, low cytotoxicity, and good tolerability with the absence of associated resistance. CI is an immobilized iodine molecule in a hydrophilic modified-starch polymer bead with the dual property of cleansing the wound by absorbing the exudate and bactericidal effect by sustained release of iodine molecules over the infected wound. The preparations comprising PI and CI improve wound healing and minimize the bacterial infestation or contamination in various chronic wounds, burns, and ulcers. This review narrates the comparison of CI and PI for the management of wounds in the context of biofilm reduction, wound size reduction, and granulation tissue promotion.

## Introduction and background

Wound care, irrespective of being acute or chronic, is a serious public health issue that impacts a large number of people with various types of wounds and costs a large number of resources. Wound care has been found to be a considerable burden on patients, healthcare providers, and the healthcare system as a whole. Wound care is a crucial factor to consider while caring for individuals with chronic wounds on a daily basis [1].

Iodine has been used as one of the most effective antiseptics for reducing infection sequelae for over a century, and topical iodine formulations have been employed for wound care. Lugol's solution is the most basic form of iodine, and it possesses stinging and caustic properties.

Infection management has long been recognized as a necessity in the treatment of wounds. During the last two decades, there have been numerous advances in the science of wound management. Wound management technologies, research, and the formulation of standards of care based on research and clinical data have all contributed to a positive outcome in wound healing. Current literature, on the other hand, supports the use of topical wound treatments for wound care and management [2].

There are several topical products available, each with its own set of benefits and drawbacks. For example, iodine-based preparations tend to release free iodine when they encounter wound exudates, acting as an antiseptic and controlling infection, aiding wound healing [3].

To overcome the drawback, iodine complexes such as povidone iodine (PI) and cadexomer iodine (CI) are used. PI is a triiodide and polyvinylpyrrolidone combination. When the paste is absorbed in wound exudates, triiodide is liberated, maintaining the balance between triiodide and the PI complex.

Iodine molecules are trapped in a hydrophilic modified-starch polymer bead called CI. The beads of polymers in CI are swelled up by the wound exudates and gently discharge integrated iodine, preventing its accumulation and thereby preventing iodine-related complications such as allergic contact dermatitis, systemic acidosis, and so on [4-6]. CI ointment showed positive effects on the increased rate of epithelial regeneration, wound contractility, desloughing, formation of granulation tissue, and neovascularization [7].

Keeping this context, we present this narrative review to compare the outcome of PI ointment and CI ointment for wound management and overcome the limitations of conventionally used PI ointment in terms of desloughing agent, increased epithelial regeneration, promotion of granulation tissue formation, wound contractility, and neovascularization.

## Review

Povidone iodine: role in wound management

In PI, iodine forms a combination with the synthetic carrier polymer povidone, which has no microbicidal action. The PI complex releases free iodine into solution in an aqueous media, and a balance is maintained, with more free iodine being liberated from the PI reservoir as the iodine-consuming germicidal activity progresses [8]. The formulation, concentration, and temperature-dependent equilibrium of povidone-bound iodine to free iodine serves as protection against the suppression of granulation tissue formation and minimizes safety and acceptability issues related to skin exposure to previous elemental iodine formulations [9,10].

Iodine's microbicidal activity involves the inhibition of critical bacterial cellular mechanisms and structures, as well as the oxidation of nucleotides, fatty/amino acids in bacterial cell membranes, and cytosolic enzymes involved in the respiratory chain, resulting in their denaturant and deactivation. At the molecular level, however, the exact sequence of events remains unclear [11].

In vitro evidence suggests that iodine not only has broad antibacterial properties but also inhibits inflammation caused by infections and the host response. These anti-inflammatory impacts are likely to be multifaceted and clinically significant [12].

Spectrum of Activity

PI is a topical antibiotic that acts against bacteria, viruses, fungus, spores, protozoa, and amoebic cysts [10]. In conventional antimicrobial testing, PI is seen to kill a number of bacteria strains found to cause nosocomial infections, including methicillin-resistant *Staphylococcus aureus* (MRSA) and other antibiotic-resistant strains, within 20-30 seconds of exposure. Comparators like chlorhexidine, on the other hand, entail considerably longer exposure times, and most species retain residual bacteria [13,14]. Because of these properties of PI ointment, it is mostly recommended for ulcerative wounds, small burns, and traumatic skin loss [15].

Resistance and Cross-Resistance

Resistance to topical and systemic antibiotics like mupirocin, fusidic acid, and gentamicin is on the rise worldwide, as is the prevalence of hospital and community-acquired illnesses. This is a serious medical problem that appears to be caused in part by the overdose and misuse of antibiotics. Clinical strains provide the majority of evidence for bacterial resistance and cross-resistance to antiseptics such chlorhexidine, quaternary ammonium salts, silver, and triclosan [8,9]. There appears to be evidence of antiseptic and antibiotic cross-resistance [14]. Systematic testing has found no resistance to PI yet. Iodine has demonstrated no acquired resistance or cross-resistance in over 150 years of use, unlike other antiseptics (with the exception of an apparent lack of cross-resistance to silver). This lack of resistance is most likely due to iodine's different mechanisms of action [16,17]. The antiseptic resistance of 20 bacterial strains was found by Yasuda et al. and was noted that PI killed all bacteria within 20 seconds [14].

Activity Against Biofilms

Biofilm is a complex microbiome structure that adheres to the surface and contains different bacterial colonies or single types of cells in a group. These cells, which are embedded in extracellular polymeric substances, a matrix made up primarily of environmental DNA, proteins, and polysaccharides, demonstrated high antibiotic resistance [18]. Iodine solutions are found to get deactivated in the presence of pus, slough, necrotic tissue, and in presence of organic materials present over the wound [19]. In the presence of antimicrobial therapies, biofilms have been shown to slow wound healing and enhance bacterial survival. PI's long-term efficacy on wound healing in the presence of biofilms was tested in a recent study. PI's in vitro efficacy against *Staphylococcus epidermidis* and *Staphylococcus aureus* growth, as well as the prevention of staphylococcal biofilm formation at sub-inhibitory concentrations, has been verified in studies. Biofilm clearance was higher in this assay than with polyhexamethylene biguanide (PHMB), octenidine, chlorhexidine, mupirocin, and fusidic acid, for example [20].

Cadexomer iodine: role in wound management

Cadexomer is a 0.9% w/w iodine-containing hydrophilic starch polymer bead. CI releases free iodine (an antiseptic) when it comes into contact with wound exudates, according to a pharmacodynamic study [3]. The product has a dual action: cleansing of the wound and bactericidal action.

It also absorbs fluid (up to 6 milliliter/gram of CI) leading to exudate management of the wound by making desloughing action easier by eliminating pus and debris. The physical swelling of the cadexomer beads while in contact with exudative fluid leads to sustained release of iodine particles leading to a bactericidal effect within the dressing for up to 72 hours. It promotes rapid granulation tissue development and accelerates wound healing with substantially less pain and edema [21-23]. Moreover, CI maintains a moist environment to aid in the healing of chronic skin lesions.

Spectrum of Activity

Within six or eight weeks of therapy, significant decreases in wound pathogens were observed with CI ointment intervention compared to standard of care (SOC) in randomized controlled trials (RCTs) in cases of venous leg ulcer patients. Hillström [24] used semi-quantitative approaches to show a substantial decline in *Staphylococcus aureus* (P = 0.001) and an improvement in infection. According to Lindsay et al., in the majority of instances, CI ointment treatment resulted in the removal or reduction of organisms, which was linked to decreased odor and ulcer improvement [25]. Skog et al. [26] reported a significant reduction in streptococci, enterococci, and *Enterobacteriaceae* such as *Proteus* and *Klebsiella* species (P < 0.0001 and P < 0.01, respectively). Thus, CI ointment is advised mainly for chronic exudative wounds like diabetic foot ulcers, venous ulcers, and pressure ulcers where slough, infection, or the risk of infection is a concern [15].

Activity Against Biofilm

Biofilms have recently been identified as a source of chronic wound infections and a delay in wound healing, with biofilms found in over 78% of chronic non-healing wounds like venous leg ulcers, diabetic foot ulcers, and pressure ulcers [27]. Biofilm provides a substantial clinical challenge, especially because of its increased antimicrobial tolerance and capacity to elude the human immune response. In vitro models combining several therapeutically relevant circumstances and substrates, such as porcine tissue, have shown that CI ointment is effective against mature biofilms; in addition, animal models indicate encouraging outcomes with CI ointment against biofilm in a wound [28].

Wound Area Reduction and Wound Healing

Following CI ointment management, 10 RCTs in the published literature showed significant improvements in wound area reduction or full healing events in chronic wounds. Hillström [24] observed a substantial reduction in wound area with CI ointment after only one week of treatment, which lasted until the completion of the research (six weeks). Furthermore, Skog et al. [26] found that the significant reduction in ulcer size by one and two weeks (P = 0.005) translated to a 34% reduction in ulcer size by six weeks, compared to a 5% increase in ulcer size in the SOC group.

Critical evaluation

While PI is most commonly recommended for ulcerative wounds, small burns, and traumatic skin loss, CI is administered in chronic exudative wounds like diabetic foot ulcers, venous ulcers, and pressure ulcers where slough, infection, or the risk of infection is a concern, according to multiple studies [15].

The findings of various in vivo investigations show unequivocally that there is sufficient evidence to recommend the use of CI in chronic wounds. According to a review, there is insufficient evidence to demonstrate that CI has a deleterious effect on wound healing and infection [21-27].

PI, on the other hand, showed to be efficient in reducing bacteria counts and preventing wound infections when used in the presence of infection in acute wounds like traumatic lacerations and burn wounds [29-31]. PI inhibits collagen formation, is toxic to fibroblasts and keratinocytes, and inhibits epithelial cell migration, thereby impairing the healing process in non-infected human wounds [9,32-34].

In human clinical studies, CI was found to be the only drug that reduced total microbial burden, including biofilm, to a satisfactory and effective level along with accelerating epithelisation and granulation tissue formation [25,35].

Reactive Equivalence of Iodine Present in PI Sugar Ointment and CI Ointment

The optimal iodine concentration for antibacterial activity was 0.01 w/v %, indicating that iodine-L-tyrosine reactivity is closely linked to antimicrobial action. The concentration of free iodine is unrelated to the concentration of total iodine, demonstrating that free but not total iodine is required for antibacterial activity. It is recommended that the concentration of iodine be increased to 0.1 w/v % because a large quantity of iodine is absorbed when used topically at low concentrations (0.01 w/v %) in wounds.

PI sugar ointment interacted more efficiently with L-tyrosine at this therapeutically relevant dose as compared to lecithin, whereas CI ointment reacted more efficiently with lecithin as compared to L-tyrosine. The quantity of iodine in PI sugar ointment that reacted with actual wound exudates was two times higher than the amount of iodine in CI ointment that reacted with wound exudates, alluding that iodine is quickly consumed by the protein component in PI sugar ointment and the antiseptic effect is quickly diminished. Iodine in CI ointment maybe eventually absorbed by protein components, allowing the antimicrobial properties to last longer [36].

Equivalence of Water Absorption Among PI Sugar Ointment and CI Ointment

Using an agarose gel to measure water absorption capacity, it was discovered that CI ointment had a 2.9-fold higher water absorption capacity per weight over 24 hours than PI sugar ointment. These findings imply that the amount of water absorbed is affected by whether the base ointment stays in its original form or dissolves after water absorption. When PI sugar ointment and CI ointment are utilized to treat pressure ulcers, PI sugar ointment may have transitory low water absorption since the base ointment gets dissolved, whereas CI ointment may have persistent water absorption as the rate-determining step is the diffusion of dissolved macrogol and water to macromolecular beads [37].

## Conclusions

A combination of PI and CI enhances healing and reduces bacterial contamination in a variety of chronic wounds, burns, and ulcers. Despite the antibacterial benefits acquired through its use, various possible downsides have been reported in its therapeutic application, with varying and contradictory findings, prompting practitioners to be cautious about using forms of iodine for topical wound treatment. Furthermore, the ability of the base ointments used in PI sugar ointment and CI ointment to absorb water differs. As a result, when these two ointments are administered for pathogenetically similar conditions, they may provide different effects. The literature has revealed that CI is more effective in treating chronic wounds, especially with high levels of exudates as compared to PI. Several studies have strongly supported that CI has a better outcome than PI with respect to desloughing action of pus and debris, reduction of wound size, and promotion of granulation tissue formation leading to better wound healing and management.
